# Hospital and Institutional News

**Published:** 1920-03-27

**Authors:** 


### HOSPITAL AND INSTITUTIONAL NEWS.

### INFANT MORTALITY.

The good effect produced by the many hygiene campaigns started in this country during recent times is reflected in the latest returns of birth and infant mortality rates, although due account must be taken of the more settled state of the country. Among other figures, we note that the births registered in the fourth quarter of 1919 were 48,202 more than in the preceding quarter, and 61,794 more than in the fourth quarter of the preceding year. It is also noteworthy that the natural increase of population in England and Wales by excess of births over deaths was 108,809, as compared with 46,977 and 40,947 in the fourth quarters of 1916 and 1917, and with a natural decrease of 79,443 in the fourth quarter of 1918. Deaths were actually 126,458 fewer than in the fourth quarter of 1918. Infant mortality was 71 per 1,000 registered births, which is the lowest recorded rate for any fourth quarter of the year. With regard to the first comparisons, which form mainly a survey of adult conditions, two great factors must be remembered—war and influenza; but with regard to the last, the infant life consideration, great credit is undoubtedly due to the improved health supervision provided.

### REPRESENTATIVE MEDICINE.

We understand that the Council of the Royal Society of Medicine decided at its meeting on March 16 officially to appoint its representatives to the Federation of Medicine and Allied Societies.

### THE "PALL MALL" PANACEA.

While the *Pall Mall Gazette* was proclaiming its panacea for the hospital "crisis," namely, the creation and extension of pay hospitals for the luckless middle classes, one such hospital at least had the sense to call attention to itself. The Florence Nightingale Hospital for Gentlewomen reminded the public, through *The Times*, that it had long been doing work which was at last asserted to be important, and thus showed that the voluntary system is neither bankrupt in ideas nor in example. But why was the Florence Nightingale Hospital for Gentlewomen alone in this matter? It is not the only institution of its kind. Surely there was no need for Fitzroy House, in Fitzroy Square, the headquarters of the Home Hospitals' Association, with its forty-two years of good work behind it to keep silent. Does it not do, and was it not founded to do, the very work which is now demanded for our salvation? The committee of Fitzroy House might well have supplemented the statement of Sir John Macalister, which referred to the cubicles at Guy's, the wing for paying patients at St. Thomas's and St. Chad's, Birmingham, by a modest mention of itself. We decline to complete the list. It is the duty of the institutions to do so. The hospital for paying patients of moderate means is an established part of the voluntary system. We have only to develop existing resources. Our *latest* recommendation of this policy was made in March and in August 1913. At last the recommendation is being taken seriously.

### THE INTERNATIONAL RED CROSS.

The recent meetings of the General Council of the League of Red Cross Societies at Geneva seem to show that it is easier to start a League of Red Cross Societies than a League of Nations. The League exists to encourage a voluntary Red Cross Society in every country for the improvement of the public health and the prevention of disease, to disseminate new medical knowledge, and to co-ordinate relief work in national emergencies. Without indulging in exaggerated hopes, we believe that the League will be a very useful international body, will act as a new bond where bonds are badly needed, and will serve, if indirectly, to maintain the Red Cross organisation, and thereby become a new buttress for the voluntary system in this country.

### THE SCHOOL DOCTOR ON LEEDS CHILDREN.

Dr. Algernon Wear, chief school medical officer at Leeds, has been urging the case for a children's hospital. He had, he said, a staff of thirty-four people to find out what was wrong with the school children; but when this had been discovered the staff could only offer advice, because there was no children's hospital for them to enter. Another speaker declared that the existence of children's wards in general hospitals was no answer to the general argument, because the children should have a hospital of their own. Leeds is so full of hospital questions at present that it would be rash to support without full consideration the ambitious plan here proposed. But we are glad to note that the conscience of Leeds has been quickened of late by the revelation of facts which, *because they are not exceptional*, are generally disguised. These facts are a disgrace to civilisation. People should be ashamed not to make them known. Therefore, whether a children's hospital be immediately practicable or not, Dr. Wear and his supporters have done well to proclaim the need for it.

### THE OPTIMIST AND THE PESSIMIST.

Mr. G. Verity, the chairman of Charing Cross Hospital, refuses to look on the black side of things, and maintains that the voluntary system has not broken down, in spite of the lachrymose jeremiads everywhere to be heard. At the Council meeting he narrated the surprising financial feat of the hospital in freeing itself from its heavy mortgage and in opening the closed wards. In spite of the increase in expenditure from £24,000 to £51,000, he has every faith in the public to see the institution through the coming bad times. On the other hand, Sir Frederick Macmillan, the chairman of the National Hospital for the Paralysed and Epileptic, did not sound so hopeful a note when announcing that the hospital (or the main part of it) would be closed on December 31 unless help was forthcoming to extricate it from the financial quagmire. But the National is so very well known and valued that we cannot believe that such a calamity as closing the whole of the wards can be allowed to eventuate. We understand that Sir Frederick's appeal for a special maintenance fund, to which promises are asked for support spread over three years, has already secured some substantial support. The needs of the oldest and largest of the special hospitals of its class have only to be widely advertised and we are confident that sufficient help to weather the storm will quickly be forthcoming.

### THE LEPER PROBLEM IN INDIA.

The movement for ameliorating the condition of lepers in India has received the support of many Indians, but more co-operation is needed in order that the problem may be adequately dealt with. A conference at which the question was discussed in all its bearings opened at Calcutta on February 3. This gathering was arranged by the Mission to Lepers. The Lepers Act, as it at present stands, does not give adequate power for segregating pauper lepers, and it is hoped that it may be so amended as to benefit the unfortunate sufferers and to protect the general community. Considering that there are some 150,000 lepers in India the matter is of urgent importance, and the movement certainly deserves the support of all who are concerned in the welfare of India.

### COCAINE HABIT IN BENGAL.

The cocaine habit, especially among young men, was giving rise to serious alarm in all the more thickly-populated parts of India before the war. The drug was smuggled into Calcutta by Austrian and German steamers trading to that port, but, of course, the outbreak of hostilities shut off these sources of supply. Cocaine of Japanese origin has now appeared on the market, and the Commissioner of Excise says there are signs that the smugglers exchange cocaine for opium.

### LORD KNUTSFORD ON CO-ORDINATION.

In an interview on the Red Cross scheme for a survey of the voluntary hospitals with a view to their better co-ordination, Lord Knutsford gave an instance of the need. At the London Hospital, he said, all our beds are full, and we have 868 people waiting. Across the road there are 200 empty beds belonging to the State in Whitechapel Infirmary, and we may not send patients there. Why not, the layman may ask? Because State control does not allow it; and this is the machine we are invited to worship to-day.

### A CHEERFUL LESSON FROM BELFAST.

We were glad to read of the success which has attended the Royal Victoria Hospital, Belfast, in the matter of employers' subscriptions. During the war two members of the committee visited every firm in Belfast; each industry was examined in turn, and the head of the Company asked to give an increased subscription. Lady Pirrie, the hospital's President, reports that in the majority of cases an annual subscription *for the first time* was forthcoming, and simultaneously a weekly collection from the employees was generally arranged. A very valuable permanent accession of income has been secured. Here, again, immense fruits have rewarded a methodical cultivation of familiar sources of revenue. Is the same method applied by London hospital committees to city firms and shops in their respective neighbourhoods? We ask and ask, but are doubtful about the answer.

### OVERCROWDING IN FURNITURE.

The appeal of the Metropolitan Hospital is noteworthy because it asks not merely for gifts of money, but for furniture and articles of value; and there is an office, 98 Stamford Hill, N., to which these gifts can be sent. It will be interesting to know what response is made to this suggestion. Almost everyone who has any furniture at all, has far too much, and spoils the effect of his house by overcrowding. Since people have many claims upon their purse, but none upon their furniture or valuables, the Metropolitan Hospital offers them a simple means of ridding themselves of superfluous "effects" to the advantage of their homes and of the hospital. What is an overcrowded room? It is a room in which the objects cannot be counted easily. To improve its appearance a clean sweep of unnecessary contents is all that is required.

### NO NONSENSE AT NEWPORT!

Mr. Peter Wright, the Mayor of Newport, deserves to be congratulated on his enterprise on behalf of the Royal Gwent Hospital there. At a meeting of the committee of the Workmen's Fund (which last year collected £2,000 for the hospital), Mr. Wright explained the improvements which were needed. He said that these were expected to cost £200,000, a sum which he was determined to raise during the current year. There are, he said, 50,000 workmen in Newport. If each of them will give one pound, he said, a quarter of the sum will be raised, and, that done, will turn for the rest to other sections of the community. A special committee was immediately formed. Several subscriptions of one pound were handed to the Mayor then and there. It is really a very fine start. Why should it be thought impossible to raise larger sums by similar means in London? If £200,000 can be raised in Newport, at least a million could be raised in the City. It is foolish to be alarmed at big figures; they are the fashion, and large sums can be raised as easily as small ones by people who understand that large and small are merely relative terms.

### AN OCTOGENARIAN'S GENEROSITY.

A successful year has attended the Crewe Memorial Cottage Hospital, which, in spite of the increased cost of maintenance, had a balance on the right side at the end of the year. In addition to this, Mr. Ralph Brocklebank has sent to the hospital a cheque for £500 to celebrate his eightieth birthday. Such good news as this is not to be slighted in a moment of popular panic.

### WOMEN IN HOSPITAL LIFE.

The Kidderminster Infirmary and Children's Hospital has appointed Mrs. Arthur Jones to be its President, and Miss Wilkinson House Surgeon. The duties of the latter post during the war are stated to have been performed or partly performed by the matron. It is a pity that no authentic record exists of the work of matron's and sisters during the war outside the strict limits of nursing. What has been done should be known, but in the absence of a careful record no inferences can be based upon it.

### PERSONAL SERVICE PENALISED.

On the occasion of their leaving England to begin work in Australia, Mr. N. G. Bolton, Hon. Secretary, and Mr. D. Williams, Hon. Financial Secretary to the Leyton War Memorial Hospital, were presented with gifts and addresses in recognition of their valuable work. The presentation took place on Monday, March 1, and was made on behalf of the subscribers by Councillor F. M. Read, J.P., Chairman of the Leyton Urban District Council. After speaking of the good and honorary work performed by the secretaries, he expressed regret that Mr. Bolton's employers, thinking that he had given too much time to the hospital, decided to dispense with his services. It seemed very hard that an employee should be penalised for his personal service in a public spirited cause. The speaker was convinced, after careful inquiry, that there had been no laxity on Mr. Bolton's part. Under these circumstances the presentation is a real tribute, and we hope that the circumstances are recorded in the address, as an inspiration to all who read it.

### FINANCE AND PERSONAL SERVICE.

The Walthamstow Dispensary, which started with a balance of £83 at the beginning of the financial year, has been able to increase this credit balance to £106. The report shows this increase to be mainly due to additional gifts and donations, but the satisfactory financial condition also reflects credit on Mr. Theo Godlee, who for twenty years has held the position of Hon. Secretary. His brother filled the post before him for no fewer than twenty-six years.

### CLAYTON HOSPITAL'S NEW SECRETARY.

Mr. Henry Maw, Assistant Secretary of the Bradford Royal Infirmary, has been appointed General Secretary of the Clayton Hospital, Wakefield. Mr. Maw has been at Bradford for the past nine years.

### A HOSPITAL LEAGUE AT TORQUAY.

We are glad to see that a Hospital League is likely to be formed at Torquay in connection with the Torbay Hospital. The idea is put forward by Mr. E. J. V. Husey, the honorary secretary of the hospital, to increase the number of its active supporters. He wishes the league to have several branches, to include the Chamber of Commerce, the trade unions, comrades, discharged soldiers and sailors, with district or ward branches, in which, we hope, the residents, far too few of whom are annual subscribers, will be enrolled. It is an excellent idea, especially suited to a place of the stamp of Torquay or Bournemouth, and Mr. Husey will earn the thanks of the hospital world if he can prove that it can be carried out successfully. In Mr. H. J. Packe, the secretary, who knows Torquay well and has worked long for the hospital, Mr. Husey will find, we hope, another enthusiast. We should like to hear how the scheme goes, for the hospital which makes it a success will have done pioneer work of real value.

### REGISTRARS OF BIRTHS AND DEATHS AND THE INCREASED COST OF LIVING.

The Ministry of Health has notified the Lambeth Board of Guardians that there is no legal authority for the payment by them of any sum in excess of the fees fixed by statute to registrars of births and deaths, and that this legal position is not affected by a resort to arbitration, which must be regarded as an invitation to an independent person to assist the parties with advice by determining the amount of any gratuity which, subject to the sanction of the department, may reasonably be awarded to registrars by the guardians. But the Minister intimated in a circular letter that he was prepared to sanction the payment of gratuities to the registrars where boards of guardians were willing to make such payments. In view of this, the Lambeth Guardians have decided to grant gratuities to their registrars calculated on the amount of fees paid to them during the year ending September last.

### ACTON COTTAGE HOSPITAL EXTENSIONS.

Large extensions are about to be made to the Acton Cottage Hospital, to cost £12,000. They include the building of two new wards and the provision of an out-patient department on the ground floor. This will comprise seats for sixty, a consulting-room, dispensary, dressing-room, two rest-rooms and water closets. The beds in the general wards will be increased from twenty to thirty-four, children's cots from eight to twelve, and the present two private wards will be increased to four. The scheme is part of a larger extension which it is hoped to complete later. The Chairman of the hospital, Mr. E. F. Hunt, suggests that the new accommodation will, if necessary, be ample for the local children's clinic, and has advised the Acton District Council to use it as such, a cheaper arrangement than building a new clinic. The new hospital accommodation is badly needed, for Acton's population in ten years has increased from 56,000 to nearly 70,000, whilst the nearest general hospital is the West London at Hammersmith.

### HOME FOR LAST-STAGE CONSUMPTIVES.

Deptford Borough Council is able to report some progress with a scheme for the provision of a home for the segregation of advanced cases of tuberculosis where patients can have every comfort, and at the same time can be prevented from being a source of danger to others. The Council's idea was for a joint establishment, and now the adjoining boroughs of Greenwich, Lewisham and

Woolwich have agreed to the principle, and so the proposal is likely to mature in due course. It has, however, been arranged at a conference of the four boroughs that accommodation shall not only be provided for persons in the later stages of disease, but also for the reception of less acute cases pending other accommodation being secured for them. It is proposed to rent four empty houses abutting on Greenwich Park for conversion into a home for about eighty patients. The cost ought not to be a burden on the local ratepayers inasmuch as the London Insurance Committee has already agreed to pay two guineas a week for every insured patient, whilst the London County Council will pay a similar sum in respect of non-insured cases.

### A TEST OF MUNICIPAL HOSPITAL SCHEMES.

The Willesden Urban District Council has already a small municipal hospital, and a scheme for extension has been prepared. The suggestion is to add 186 beds, and, at present-day prices, the cost of the extension is said to be about a quarter of a million, whilst the maintenance of the extension will cost about £40,000, though £7,644 of this would be recoverable in Government grants. The Council's Health Committee now reports this scheme is so extensive that more State aid should be secured, and accordingly the medical officer of health has been instructed to communciate with the Minister of Health, to lay the Council's difficulties before him. If they were also laid before the ratepayers, the real popularity of muncipal hospitals could be judged.

### INCREASE OF MATERNITY CASES.

Acting on the advice of Dr. H. W. Bruce, medical superintendent at the Southwark Infirmary, the guardians have decided to provide a properly-equipped maternity ward for lying-in cases at the infirmary. Dr. Bruce pointed out that in five months there had been fifteen births in the infirmary, the numbers being greatly in excess of any that have ruled in the past. Most were complicated cases, and it was impossible to treat them properly under existing conditions. By a slight reorganisation, however, and the removal of a few chronic cases to Newington Institution, a maternity ward could be organised. The accommodation thus provided would be sufficient for all the cases, whilst provision could be made to treat emergency cases at Newington Institution, where a midwifery nurse could be in attendance. The new arrangement should be a great boon to the medical and nursing staffs as well as to the patients.

### PROPOSED COUNTY HOSPITAL FOR RUTLAND.

At the annual meeting of the Oakham Cottage Hospital, a suggestion for the establishment of a county hospital was considered, and on the motion of the Hon. Mrs. Gretton a joint committee was appointed to make the necessary arrangements for co-operation. Mrs. Gretton reported that £3,245 had already been promised for the proposed new hospital, of which £500 was a grant from the Royal Red Cross Society in London. Other funds would be forthcoming, including about £170 from the Prisoners of War Fund. Lord Lonsdale had again made the offer of a site for the new building, and there was a good prospect of the proposal eventuating. The Cottage Hospital, which had been closed since June 1, 1917, owing to the difficulty of obtaining a staff, had been reopened, and the Hon. Treasurer reported total receipts £745, expenditure £706. The lease of the hospital expires in 1924.

### CINEMA IN HOSPITAL.

Two thoughts arise from a cinema entertainment that was lately given at the Royal Sussex County Hospital at Brighton in regard to the subject of the pictures; those shown at Brighton were selected with taste; but there are many pictures which might unduly excite a patient. As regards length of entertainment, this particular display lasted two hours, during which four films were shown. This sounds rather a long time. However, the innovation at Brighton was thoroughly appreciated by the patients.

### SHOULD DISTRICT MEDICAL OFFICERS BE ABOLISHED?

Lambeth Board of Guardians has decided to extend the present system of dealing with the outside poor, which has been in vogue for some years in the inner wards of the borough. This scheme does away with the district medical officers, and places the whole treatment of the poor under the medical staff of the infirmary, whose members visit at stipulated times the various dispensary centres. All cases of extreme sudden illness are reported direct to the medical superintendent at the infirmary, who either visits the case himself or asks one of his staff to do so. From every point of view this arrangement seems to be satisfactory. For not only in Lambeth, but also in other large London boroughs, delay often through no fault of his own used to occur before a district medical officer reached a patient, but under the new arrangement this difficulty is removed. Hence the complaints of inefficiency or delayed treatment do not occur.

### THIS WEEK'S DRUG MARKET.

While prices on the whole are firmly maintained there is a tendency towards lower values in several cases. Quotations for strychnine have again been advanced in consequence of the rise in the price of nux vomica. Santonin is very scarce and extreme prices are asked for anything available. The high price of ipecacuanha is firmly maintained. The demand for menthol has improved and the price has moved upwards again. Japanese refined camphor also inclines upwards in value. Cod-liver oil after moving downwards in price has a strong upward tendency. German bromide of potassium is more freely available. Citric acid keeps at its high price and tartaric acid is dearer in sympathy. Phenacetin and phenazone still tend upwards in value. Lemon, orange, and bergamot oils are quoted at higher prices. Value of salicylic acid is not so firmly maintained. Chamomiles are offered at rather lower rates and Japanese peppermint oil is cheaper.

